# Ultrasonic diagnosis of asymptomatic rupture of uterine in second trimester of pregnancy after laparoscopic surgery for interstitial pregnancy: a case report

**DOI:** 10.1186/s12884-021-03845-y

**Published:** 2021-05-14

**Authors:** Chun Tong, Lijun Gong, Yuan Wei, Zhaohui Liu, Yiting Wang, Pengbo Yuan

**Affiliations:** Department of Obstetrics and Gynecology, Peking University Third Hospital, 49 North Garden Rd, Haidian District, Beijing, 100191 China

**Keywords:** Ultrasound, Uterine rupture, Interstitial pregnancy, Asymptomatic

## Abstract

**Background:**

Uterine rupture is a rare, life-threatening event in obstetrics that may be fatal for the mother and fetus. Therefore, obstetricians need to pay attention to and should consider the antenatal diagnosis of uterine rupture in women having its risk factors. Successful conservative management for asymptomatic uterine rupture due to previous laparoscopic surgery for interstitial pregnancy has already been reported but remains understudied.

**Case presentation:**

A 39-year-old woman was diagnosed asymptomatic uterine rupture at 22 weeks gestation by a routine second-trimester ultrasound scan. She had a history of laparoscopic salpingectomy with cornual wedge resection for interstitial pregnancy 10 months before this pregnancy. Refusing doctor’s twice advice of terminating the pregnancy, the patient insisted carrying on the pregnancy, and followed up by ultrasound and magnetic resonance imaging. Fetal growth was appropriate, fetal movements were good and the patient had no symptoms, without uterine contraction or amniotic fluid loss throughout follow-up period. Caesarean section was carried out at 34 + 1 weeks with a good maternal and neonatal outcome.

**Conclusions:**

A previous history of laparoscopic salpingectomy with cornual wedge resection could be a risk factor for uterine rupture in pregnant women. Sonographers should be alert to this potential risk in pregnant women with a history of laparoscopic salpingectomy with cornual wedge resection even in asymptomatic patients.

## Background

Uterine rupture is one of the most catastrophic complications of pregnancy, carrying an increased risk of maternal and perinatal morbidity and mortality. Incidence of uterine rupture was 0.03% in China [1] (including complete rupture and incomplete rupture). Abdominal pain, bleeding and non-reassuring fetal status are the common signs [2]. We report the diagnosis and treatment of an unusual case of asymptomatic uterine rupture that occurred at 22 weeks’ gestation with a history of interstitial pregnancy treated with laparoscopic salpingectomy with cornual wedge resection.

## Case presentation

**Fig. 1:**
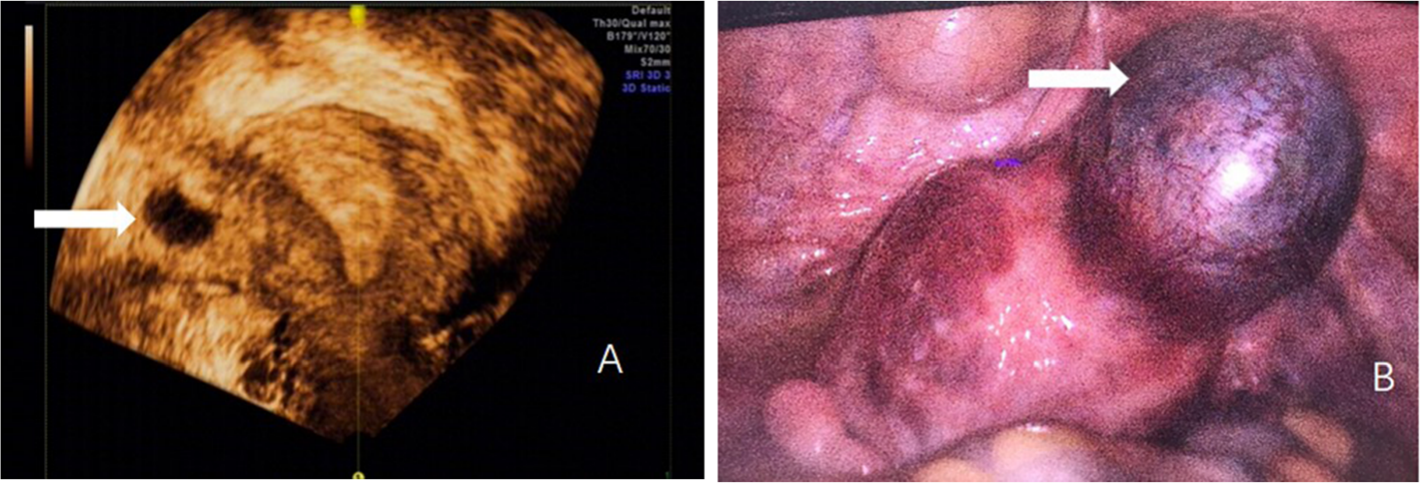
Right side interstitial pregnancy at 7 weeks gestation. **a** 3D transvaginal ultrasound imaging; **b** Laparoscopic imaging from cephalad view. Arrow illustrates right interstitial pregnancy

A 39-year-old woman, gravida 3 para 1, came to this institution at 22 weeks gestation for routine second-trimester fetal anatomical ultrasound scan, with no vaginal bleeding or any abdominal symptoms. The patient’s obstetric history included one uneventful term delivery 7 years ago and a laparoscopic surgery 10 months before current pregnancy (Fig. 1a, b). The laparoscopic salpingectomy was for a right interstitial pregnancy. The surgeon used bipolar electrocoagulation along the edge of the pregnancy capsule, cut the intestinal pregnancy lesion with scissors and stitched the incision of uterine cornual with double-layer continuous suture with 0 # Vicryl. The patient lost 20 ml blood during the surgery and was discharged on postoperative day 2 in satisfactory condition.

**Fig. 2:**
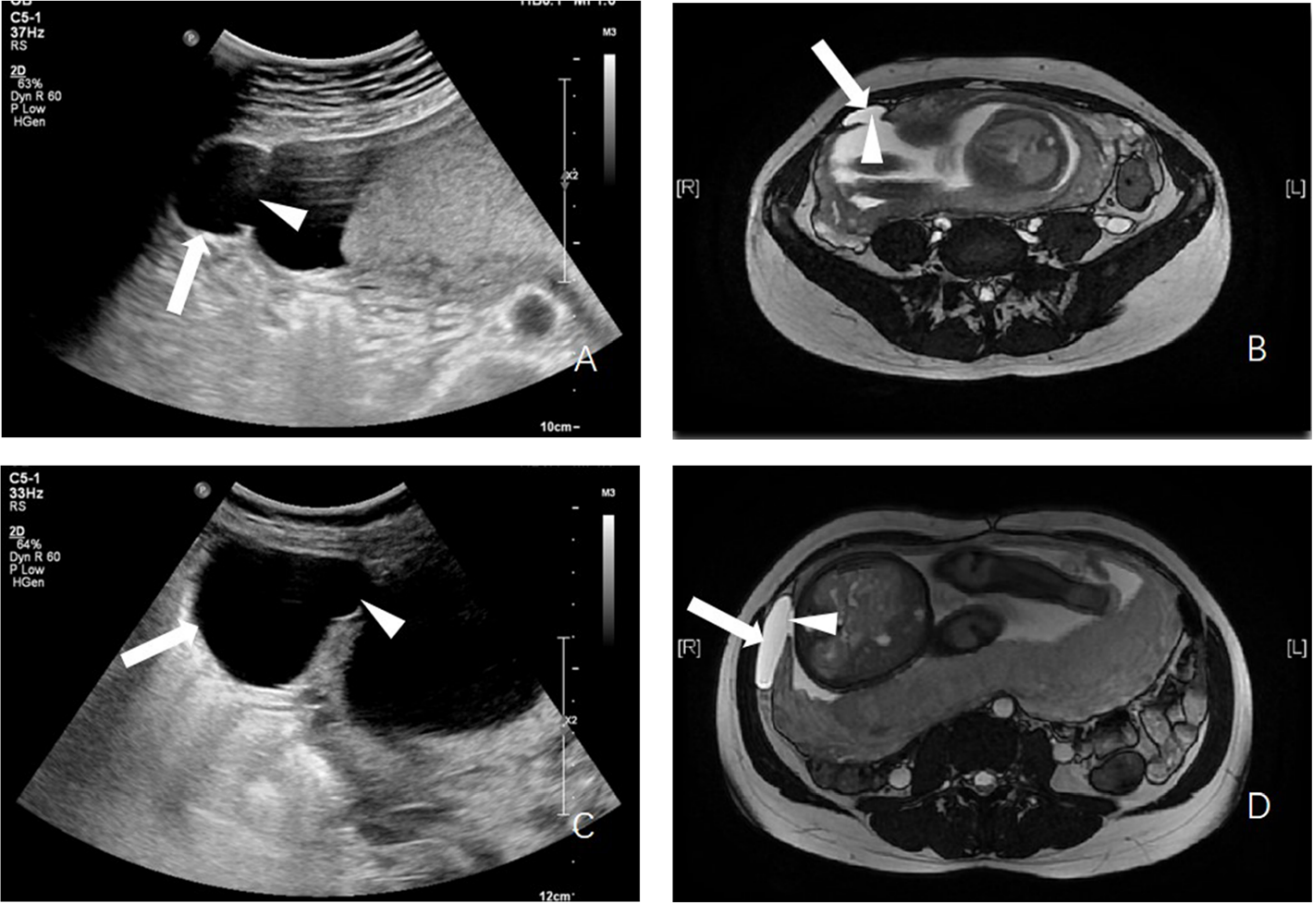
Amniotic sac containing the amniotic fluid without any fetal structures or umbilical cord herniated from a defect in the right uterine cornual into the peritoneal cavity. **a** Ultrasound imaging at 22 weeks gestation; **b** MRI imaging at 22 weeks gestation; **c** Ultrasound imaging at 32 weeks gestation; **d** MRI imaging at 32 weeks gestation. Arrow illustrates herniated amniotic sac, triangle illustrates uterine wall defect

The 22 weeks gestation ultrasonograms demonstrated normal fetal growth pattern and normal fetal anatomy. The cervix was long and closed. The amniotic sac was about 30 × 30 mm containing amniotic fluid without any fetal structures or umbilical cord was noted herniating from the defect of right uterine cornual into the peritoneal cavity (Fig. 2a). The placenta was located far away from the cornual. Free effusion was not seen in abdominal or pelvic cavity. Uterine rupture was suspected and clinicians were notified. Magnetic resonance imaging (MRI) scan was performed, which provided similar findings (Fig. 2b). The above findings supported a diagnosis of ruptured uterus and the patient was admitted to the inpatient ward.

The strong association between uterine rupture and maternal fatality is supported by multiple studies, including 6.6% by Astatikie G [2], 12% by Delafield R [3]. Based on these literatures, doctors from maternal-fetal medicine department carefully evaluated this case, and suggested termination to the patient and her husband with full disclosure of risks to the mother if the pregnancy continued. The couple decided to continue with the pregnancy and the patient returned home with medical advice of bed rest. She then took outpatient ultrasonic scan twice a week and MRI 8 weeks later.

At 30 + 5 weeks’ gestation, the patient was admitted to the hospital for preventive dexamethasone to promote fetal lung maturation. During her hospitalization, she was given continuous monitoring of the fetal heart rate and uterine contractions. By 32 weeks both ultrasound (Fig. 2c) and MRI (Fig. 2d) showed increased herniation of the amniotic sac about 70 × 50 mm. Fetal growth was appropriate with normal amniotic fluid. Considering the increased herniation, doctors suggested cesarean section despite that the patient had no symptoms and the fetal status was reassuring. The couple insisted to prolong pregnancy to 34 weeks. In the next 2 weeks, the patient had good fetal movements, no uterine contraction or amniotic fluid loss.

**Fig. 3:**
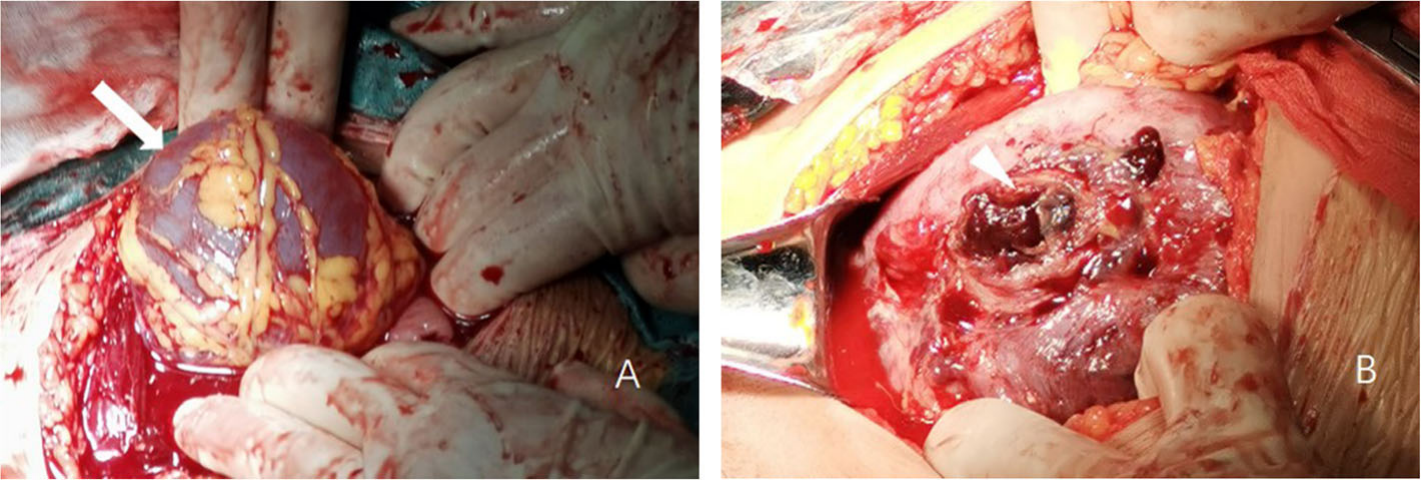
Intraoperative image. **a** Herniated amniotic sac before fetal delivery; **b** Uterine rupture after fetal delivery. Arrow illustrates herniated amniotic sac, triangle illustrates uterine wall defect

Caesarean section was performed at 34 + 1 weeks. There was no hemoperitoneum at the point of entry into the abdominal cavity. An intact amniotic sac measured 70 × 70 mm was noted herniating through a defect in the right upper fundus of the uterus, with omentum wrapped and adhered around it (Fig. 3a). A male baby weighted 2600 g was delivered by lower transverse uterine incision, with Apgar scores of 10 and 10 at 1 and 5 min, respectively. After delivery and removing the herniated amniotic sac, a 20 mm rupture was revealed in the upper portion of the right fundus (Fig. 3b). The defect was repaired using one layer of running sutures and one layer of buried vertical mattress suture with 0 # Vicryl.

Following 7 days’ postoperative monitoring the puerpera and the infant were discharged home. The patient was counseled extensively about the risks of future pregnancy.

## Discussion and conclusions

Spontaneous uterine rupture usually occurs in the presence of a uterine scar, often from a previous cesarean delivery, or myomectomy [1]. Recently there have been new, though in limited number of reports [4, 5] about uterine ruptures during subsequent pregnancy in patients with a history of laparoscopic surgery for interstitial pregnancy. Wye D et al. diagnosed uterine rupture at 30 weeks gestation after prior laparoscopic cornual wedge resection for a right sided interstitial pregnancy [4]. An emergency caesarean section resulted in a good outcome for both mother and baby. Su CF et al. diagnosed uterine rupture at the scar of prior laparoscopic cornuostomy for a left interstitial pregnancy after vaginal delivery of a full-term infant [5].

Prior studies have indicated the influence of thermal damage caused by bipolar electrocoagulation and lack of multiple-layer suturing [6, 7]. Some earlier studies looked at the relationship between interdelivery interval length and obstetric outcomes after cesarean, Bujold E et al. [8] suggested that the odds ratio for uterine rapture is 2.65 for interval < or = 24 m; while Viteri O et al. [9] suggested that no association between interdelivery interval length and adverse maternal outcomes, including uterine rupture. These reports suggest a need to further study the contributing factors of uterine rupture after laparoscopic salpingectomy with cornual wedge resection.

This case also reminded doctors and sonographers to be aware of the existence of asymptomatic uterine rupture in patients after laparoscopic salpingectomy with cornual wedge resection. Several case reports illustrated a herniated amniotic sac into the abdominal cavity being a unique initial manifestation of uterine rupture and were easily misdiagnosed as adnexal cyst.

Jinhua Dong et al. reported a double-corner uterine rupture following salpingectomy in a twin pregnancy at 28 weeks gestation [10]. Ultrasound imaging revealed a 183 mm × 112 mm anechoic zone in the right front of the uterus. It was considered as an adnexal cyst at the first time but ultrasonic 2 days later revealed that the anechoic area was connected to amnionic sac. An emergency caesarean section avoided adverse events. Wali AS et al. presented a case of an asymptomatic uterine rupture at 29+ weeks gestation with amniocele that remained misdiagnosed as large ovarian cyst on ultrasound, during subsequent 3 weeks, all the amniotic had fluid shifted in this amniocele resulting in ultimately fetal demise [11]. In this case, the key factors included the typical ultrasonic images and the sonographer had sufficient diagnostic awareness.Uterine rupture is a life-threatening event that may be fatal for the mother and fetus. Termination of pregnancy would normally be recommended when uterine rupture occurs. In this case, doctors suggested termination of pregnancy at 22w and 32w respectively, though both rejected by the patient. Despite of some existing reports of survival, it remains rare that the amniotic sac protruded from the uterus for so many days without rupturing. It is noteworthy that if the patient’s condition progressed rapidly, the consequences would be worrying.

This case suggested two points. Firstly, a previous history of laparoscopic salpingectomy with cornual wedge resection could be a risk factor for uterine rupture in pregnant women. Secondly, when performing ultrasound scans, we should raise sufficient diagnostic awareness to women with a history of laparoscopic salpingectomy with cornual wedge resection even in asymptomatic patients.

## Data Availability

The datasets used and/or analysed during the current study are available from the corresponding author on reasonable request.

## Abbreviation

MRI: Magnetic resonance imaging
